# Intervertebral Disc Progenitors: Lessons Learned from Single-Cell RNA Sequencing and the Role in Intervertebral Disc Regeneration

**DOI:** 10.3390/bioengineering10060713

**Published:** 2023-06-12

**Authors:** Yu-Dong Zhao, Yong-Can Huang, Jia-Liang Lin, Wei-Shi Li

**Affiliations:** 1 Department of Orthopaedics, Peking University Third Hospital, Beijing 100191, China; zydddg99@163.com (Y.-D.Z.); jialianglin@bjmu.edu.cn (J.-L.L.); 2 Engineering Research Center of Bone and Joint Precision Medicine, Beijing 100191, China; 3 Beijing Key Laboratory of Spinal Disease Research, Beijing 100191, China; 4 Shenzhen Engineering Laboratory of Orthopaedic Regenerative Technologies, Department of Spine Surgery, Peking University Shenzhen Hospital, Shenzhen 518036, China; y.c.huang@connect.hku.hk

**Keywords:** intervertebral disc, progenitors, single-cell RNA sequencing, regeneration

## Abstract

The tremendous personal and economic burden worldwide caused by low back pain (LBP) has been surging in recent years. While intervertebral disc degeneration (IVDD) is the leading cause of LBP and vast efforts have been made to develop effective therapies, this problem is far from being resolved, as most treatments, such as painkillers and surgeries, mainly focus on relieving the symptoms rather than reversing the cause of IVDD. However, as stem/progenitor cells possess the potential to regenerate IVD, a deeper understanding of the early development and role of these cells could help to improve the effectiveness of stem/progenitor cell therapy in treating LBP. Single-cell RNA sequencing results provide fresh insights into the heterogeneity and development patterns of IVD progenitors; additionally, we compare mesenchymal stromal cells and IVD progenitors to provide a clearer view of the optimal cell source proposed for IVD regeneration.

## 1. Introduction

Low back pain (LBP) is an increasing social and economic burden on both global governments and individuals [1]. One of the major causes of LBP is the intervertebral disc degeneration (IVDD), which is characterized by the loss and dysfunction of IVD cells and the exhaustion of IVD progenitors [2,3]. The further development of IVDD leads to disc herniation, which exacerbates LBP. Current therapies against IVDD and disc herniation mainly include immobilization, analgesic drugs and surgeries [4]. While these therapies alleviate the symptoms, none reverse the IVD condition; however, developing stem/progenitor therapies could restore the IVD matrix and promote the growth of IVD cells. The IVD consists of three parts that are from distinct embryonic origins: the nucleus pulposus (NP), the annulus fibrosus (AF) and the cartilaginous endplate (CEP). The NP originates from the notochord [5], while the AF and CEP originate from the sclerotome [6]. After a series of cellular transformations with intrinsic regulation, the notochord turns into the NP, and the maturation of cells within the AF and CEP also results in the ablation of multipotency. However, a group of progenitors within the IVD retain their stemness and may play critical roles in future therapies.

Progenitors originating from NP, AF and CEP have all been discovered, and they possess tremendous potential to revive degenerated IVDs [7] by differentiating into corresponding mature IVD cells, giving rise to local mature IVD cells, promoting IVD matrix production and modulating several signaling pathways [8]. Several stem/progenitor cells clinical experiments have already been carried out to treat IVDD. However, current clinical studies tend to focus on the use of traditional mesenchymal stromal cells, such as bone marrow stromal cells (BMSCs) [9]. Although significant progress has been made, not enough studies have been conducted to allow comparisons to be made between IVD progenitors and traditional cells; thus, we are unable to determine which cells are the optimal choice for progenitor therapy [10]. Additionally, the origins and developmental routines of IVD cells have yet to be identified, especially in terms of how to precisely manipulate IVD progenitor differentiation.

## 2. The Heterogeneity of IVD Cells: Evidence from Single-Cell RNA Sequencing

**Figure 1:**
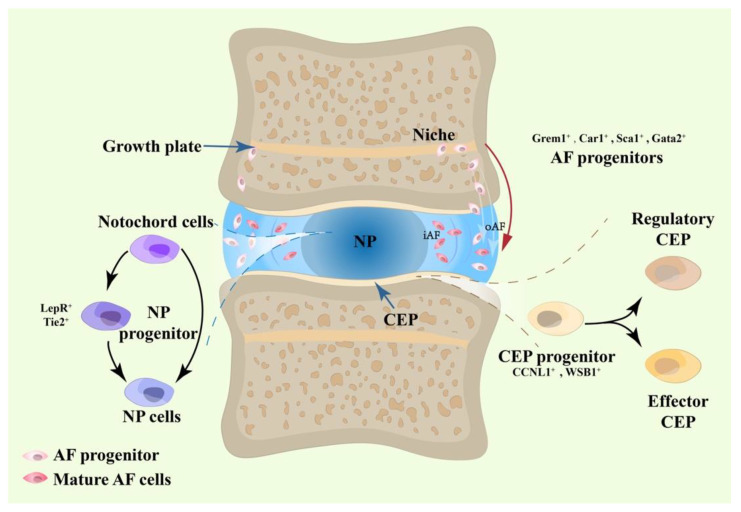
Evidence from single-cell RNA sequencing to determine the existence of IVD progenitors.

As each part of the IVD possesses distinct cell compositions, developing a deeper understanding of heterogeneity is a prerequisite for cracking the development code of the IVD cells [10]. As illustrated in Figure 1, using single-cell RNA sequencing techniques, the discovery of novel clustering patterns and progenitor markers has provided fresh insights into the existence and development of IVD progenitors. 

Table 1 summarizes the isolation protocols for the IVD progenitors, which involve mechanically mincing, digesting, expanding and confirming the phenotypes. The age of the donor is a primary factor affecting cellular biology. Tie2^+^ cells, which were thought to be potential IVD progenitors, have been found to possess much lower viability when they were isolated from older donors, and the viability decreases rapidly after the age of 25 in human donors [11]. Similar conclusions were found in the murine and canine IVD progenitors [12]. 

### 2.1. NP Progenitors

Single-cell RNA sequencing supports the existence of NP progenitors. In a recent study, uniform manifold approximation and projection (UMAP) analysis identified 15 cell populations in human neonatal and adult IVDs, within which a special cluster was termed as NC/NPC because it possessed both the notochord cell (NC) marker SOX4 and the NP cell marker Col2a1, suggesting a transition state in the development process or the presence of an NP progenitor [18]. Transcriptional entropy analysis, which evaluates the extent of stemness, was also implemented in the bovine tail IVD; by using the transcriptional entropy score of the NC cluster (0.89) as a benchmark, the possible progenitor clusters in the NP region reached a score of 0.86, while other clusters achieved lower scores [19]. Although NP progenitors were derived from NCs, it has been found that the NC–NP progenitor–NP route is not the only development route [18]. 

Single-cell RNA sequencing detected other clustering patterns. NP cells extracted from human NP tissues were classified into six clusters presenting different functions, such as immunomodulation, fibrocartilaginous growth and inflammation. CD70^+^ and CD82^+^ NP progenitors have also been found [20], and UTS2R [21] and PDGFRA [22] were identified as progenitor markers in human IVDs. In rat IVDs, stem-like cells expressing MSC markers were observed and termed NP progenitors [23]. Clusters close to notochordal lineages in bovine discs were also discovered and characterized by the pluripotent or progenitor genes KRT15, CD44 and CD55 [24]. Another study that adopted degenerated human NPs as specimens suggested the presence of NP progenitors using the leptin receptor (LepR), which has recently been identified as a stem/progenitor marker [25]. NP progenitors were found to be positive on LepR and displayed a descending trend afterbirth like NCs. Anabolic matrix proteins, such as aggrecan, also generally surrounded LepR^+^ NP cells [25].

### 2.2. AF Progenitors

Single-cell RNA sequencing revealed multiple clusters within bovine tail AF, representing various functions, including AF progenitor cells, which were found by single-cell RNA sequencing [24]. The transcriptional entropy analysis found the potential high stemness of cell clusters; the entropy score stayed around 0.85–0.86, compared to 0.89 for NCs and 0.86 for potential NP progenitors. The potential AF progenitor existed only in the outer AF (oAF), as the score of the inner AF (iAF) cells was lower than 0.85 [19]. A group of type-II collagen positive cells found in the AF contributed greatly to IVD development and repairment, presenting a descending trend afterbirth. In addition, the deletion of the type-II collagen gene led to the disruption of the spine pattern, characterized by an apparent reduction in the cartilaginous area and ECM production [26]. Another study using rat AF identified Grem1^+^ cells as AF progenitors, which was proved by their stemness markers Id1, Cripsld1, Cytl1 and Fos, as well as their high entropy scores [27]. 

The temporal and developmental patterns of AF cells were also discovered in mice models. AF progenitor cells from mice IVD were divided into five clusters based on the molecular signature, such as Car1, Adgrg1 and Cnmd. The earliest form of the AF progenitors lay in the stem cell niche adjacent to the epiphyseal plate, and they migrated through a specific route to the AF and differentiated into chondrocyte-like AF and fibroblast-like AF progenitors. Both types of progenitors developed into corresponding mature AF cells and migrated simultaneously to the inner and outer AF, respectively [17]. Notably, increasing evidence showed that AF progenitor cells exist in the outer AF (oAF) zone rather than the inner AF (iAF), as the stem/progenitor marker LepR was primarily aggregated in the oAF [25]. Moreover, bovine oAF cells had higher scores on entropy analysis [19], and type-II collagen positive cells were more intense in the oAF area [26].

### 2.3. CEP Progenitors

CEP progenitors were found to be spindle-shaped and positive in CD29, CD44, CD73, CD90 and CD105 but negative in CD34, CD45, CD11b, CD19 and HLA-DR [28,29]; novel biomarkers such as CCNL1 and WSB1 were also found to be positive in CEP progenitors [8]. Previous studies have shown that CEP progenitors have osteogenic, adipogenic and chondrogenic potential [28,30].

In a single-cell RNA sequencing utilizing human IVD data, three clusters of chondrocytes were found: homeostatic, regulatory and effector. Trajectory analysis predicted that the homeostatic chondrocytes, which express CCNL1, WSB1 and MSCs markers, were located in the root area in place of CEP progenitors; then, they became regulatory and effector chondrocytes that were responsible for IVDD and bone/cartilage growth, respectively [8].

## 3. Key Pathways in the Early Development of IVD Progenitors

Cells within the IVD possess unique developmental trajectories. During embryogenesis, the early stage of the human embryo forms the axial mesoderm and the paraxial mesoderm. The axial mesoderm then becomes the notochord, whose cells are currently thought to have a multilineage differentiation ability and maintain disc homeostasis. Notochordal cells transform into NP cells and offer migration and differentiation signals to transform the paraxial mesoderm-originated sclerotome into the AF [31]. As mentioned previously, mature discs also comprised various functional clusters that demonstrate distinct molecular signatures and prospective functions, and they derived from the corresponding IVD progenitor cells. Although mechanisms for curing IVDD have been proposed [32], many studies have solely focused on helping IVD progenitor cells survive longer in the harsh environment within the degenerated IVD or on activating their therapeutic potential [33]. However, it is important to understand the mechanisms responsible for early differentiation [32]. It is also crucial to use these mechanisms to promote the more precisely directed differentiation of IVD progenitors to reduce degeneration.

**Figure 2:**
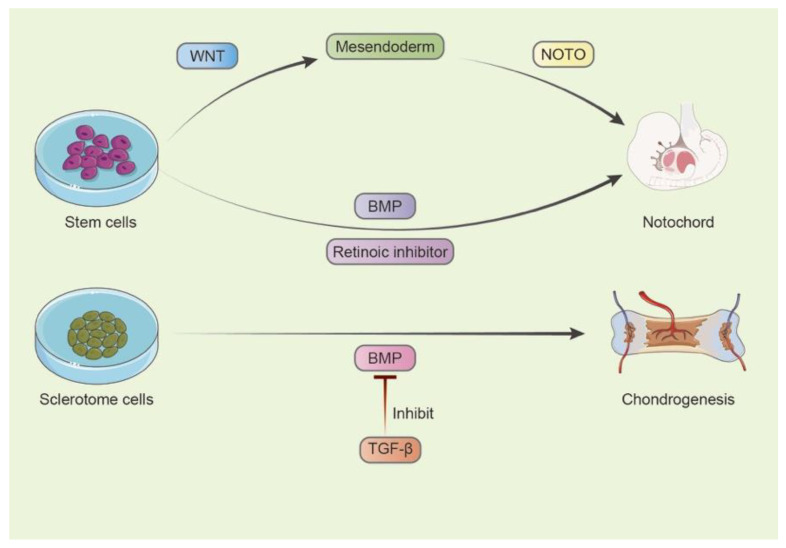
Key pathways controlling progenitor development.

As summarises in Figure 2, notochord formation involves a series of intrinsic signals, including BMP, WNT and Activin/Nodal. By activating the WNT pathway via GSK3β, CHIR definitively induced differentiation towards mesendoderm progenitor cells (MSPCs), but it was not enough to maintain their notochordal fate, even with Activin/Nodal. The subsequent transfection of the notochordal typical gene NOTO in MSPCs created and maintained a stable notochordal cell population, which was in accordance with a previous study [34]. Sequencing results coincided with this, as the prolonged expression of mesendoderm genes persisted only in NOTO-transfected cells. Additional functional enrichment analysis highlighted notochord development, which further validated this finding [31]. This finding supports the vital impression of the WNT and BMP pathways in the formation of NC cells. 

The notochord marker gene NOTO transfection in mesendoderm progenitor cells was also found to induce distinct expression genes [31]. Upregulated genes can be divided into two clusters. The maximum expression level of one cluster appeared on day 3 post-transfection and degraded on day 7, while the other peaked on day 7, including the pivotal notochord markers sonic hedgehog (Shh) and FoxA1 [31]. In another study, LAFC-induced notochord differentiation from hESCs demonstrated the decreased stemness markers Nanog and Sox-2 at day 6, which represented an effective commitment of mesodermal lineage [34]. A conclusion can be made that, in notochord induction, approximately one week might be the key point of success. 

Another study combined BMP and the retinoic acid inhibitor LDN/AGN/FGF (LAF treatment) to initiate NC differentiation, marked by an increased expression of NOTO and FOXA2 mRNA levels, which could also be enhanced by CHIR. Single-cell RNA sequencing revealed that, after LAFC (LAF + CHIR) treatment, human ESCs developed NP-like cell clusters [34]. The ablation of the type-II collagen gene in mice embryos also resulted in calcified vertebrae, short limbs and rapid death after birth, and postnatal deletion disrupted AF and NP cells and ECM formation. This experiment highlighted the importance of the type-II collagen gene, suggesting that it might be the controlling gene of progenitor function [26]. 

For sclerotome development, chondrogenesis and fibrogenesis require varied intercellular signals. TGF-β increased the expression of the BMP antagonist Noggin and thus inhibited the BMP/Smad pathway, promoting chondrogenesis. Sclerotome cells were induced into chondrocytes by signaling molecules from the BMP family; however, this process could be inhibited by TGF-β1. Additionally, BMP/Noggin enhanced Sox-9/4 and Scx expression. On the other hand, exogenous BMP inhibitors, such as Gremlin, suppressed chondrogenesis, but not enough to initiate the fibrotic differentiation of sclerotome cells [35]. This finding indicates the lineage differentiation of the sclerotome and ways to optimize progenitor/stem cell therapy. 

In short, single-cell RNA sequencing not only provided fresh information about the heterogeneity within the IVDs but also implied that important pathways (such as BMP, WNT and retinoic acid) control the development of IVD progenitors (Figure 2).

## 4. Characteristic of Disc Progenitor Cells Compared to Traditional MSCs

Bone marrow stromal cells (BMSCs) demonstrate a typical spindle-shaped fibriform outline, positive for CD29, CD90, CD105 and CD146 and negative for CD34 and CD45 [36]. BMSCs can differentiate into various IVD cell lineages, such as NP cells [37,38] and AF cells [38]. They also preserve osteogenic [36] and chondrogenic [39] capabilities. An ex vivo experiment demonstrated that BMSCs can ameliorate IVDD by preserving NP and AF cells [37,40] and can enhance matrix regeneration [38,41]. Coculturing NP cells with BMSCs led to decreased levels of type-II collagen and MMP-13, as well as increased levels of type-I collagen and aggrecan; this process was also named ECM remodeling [42]. The interaction between cells may be attributed to complex immunomodulation involving TGF-β [43]. Its therapeutic potential has been proven in rabbit [41,44], canine [39], porcine [45], sheep [46] and rat [47] models.

Umbilical cord stem cells (UCSCs) were positive for CD29, CD44, CD73, CD90, CD105 and CD166, and negative for CD11b, CD14, CD34, CD45, CD79 and HLA-DR [48,49,50]. UCSCs also possessed multilineage differentiation potential. Various in vitro studies have demonstrated that UCSCs can differentiate into all three lineages of cells: osteocytes, adipocytes and chondrocytes [49,51]. Experiment also showed that UCSCs could promote osteogenesis [48] and NP-like differentiation [52,53]. In one study, cells from Wharton’s jelly, which displayed stem cell markers, enhanced matrix production and revived degenerate NP cells [52]. In another study utilizing UCSCs, purified exosomes improved NP cell viability by adjusting the methyltransferase METTL14 [54]. The UCSCs regenerated bony connections between vertebrae, and their repairing effect extended to the AF [55], thus accelerating cartilaginous regeneration [51]. In vivo studies conducted on rabbits [53,55] and rats [51] verified the therapeutic potential of UCSCs. However, another in vivo experiment on rat models demonstrated a less satisfying outcome of the regeneration ability of human UCSCs, which may be due to heterogeneity [50]. 

Cytometry results revealed that adipose-derived stem cells (ADSCs) were positive for CD73, CD105, CD44 and Sca-1 [56] and negative for CD34, CD11b and CD45 [57]. NP-like [57] and adipocyte differentiation can be induced in ADSCs. Compared to BMSCs, ADSCs exhibited a higher potential for differentiating into NP-like cells, as shown by both genetic and mRNA analysis [56]. Staining also revealed the osteogenic and chondrogenic potential of ADSCs [58]. In addition, ADSCs were found to proliferate faster than BMSCs in both 3D and 2D cultures [56]. However, another study found that unstimulated ADSCs and BMSCs had similar proliferation abilities, which may be ascribed to different test methods. ADSCs migrated to NP-rich regions and induced a higher cell density of Tie2^+^ NP progenitors in an ex vivo degenerated ovine disc [59]. Transplanting ADSCs also revived degenerated chondrocytes and promoted endogenous repair, possibly by enhancing chondrogenic cytokines [60]. Except for the enhancement and differentiation of local cells, these multipotent stem cells were capable of restoring the extracellular matrix within the IVDs by improving the production of glycosaminoglycan (GAG) and proteoglycan [56,59]. ADSCs also exerted immunomodulatory effects, as they could produce more anti-inflammatory cytokines under inflammatory conditions [58]. The conditioned medium of ADSCs and extracellular vesicles also reduced inflammation of the AF area and exerted a protective influence [61]. Rat [56,62], sheep [63], mice [57] and rabbit [64] in vivo models were used to indicate the therapeutic potential of ADSCs. 

As shown in Table 2, the comparisons of the characteristics of IVD progenitors and MSCs are summarized. NP progenitors are fibroblast-like bipolar spindle cells that form a whirlpool array in monolayer cultures. They were positive for CD24, CD73, CD90, Tie2 [65] and CD44 and negative for CD29, CD45 [66], CD11b, CD14, CD34 and HLA-DR. Another experiment found them positive for CD29 and CD105, but with much lower amounts than UCSCs [49]. Some studies have suggested dividing NP progenitors into two groups based on their levels of MSC markers expression [67], of which aging was the major cause. The novel markers PDGFRA [8] and UTS2R [21] were also discovered. Cells harvested from human degenerated IVDs could induce osteogenesis in certain media [68], and staining revealed that calcium deposition and lipid drops could be found within those cells [49]. Chondrogenesis was also detected [69]. NP progenitors were able to produce type-II collagen and aggrecan [66], reducing the matrix loss caused by punctures [70]. Various experiments proved the multilineage differentiation ability of NP progenitors [69]. Gene analysis conducted in a recent study revealed that NP progenitors may play a key role in extracellular matrix regeneration, mineral deposition, ossification, cartilage repair and immunomodulatory reactions such as T/B cell activities [8]. The same study also suggested a complex cell-to-cell signaling cascade involving multiple immune pathways, such as SPP1 and MIF. In addition, NP progenitors produce growth factors and exert a possible supporting influence on both themselves and surrounding cells [8]. However, animal tests are far from abundant, as in vivo experiments have only been conducted on rats [70,71]. When NP progenitors were transplanted into degenerated rat IVDs, they survived in a harsh environment and facilitated ECM restoration [72,73] by increasing proteoglycan and type-II collagen and damping MMP13 expression. An increased water content and elastic modulus were also found in punctured NP injected with exogenous NP progenitors [73]. This finding supported the imaging results indicating that the disc height was better preserved after transplantation. Studies have also utilized NP progenitors pre-conditioned by biomaterials [73] or combined with scaffolds [74] for potential future applications, but none of these studies tested the migration ability of NP progenitors. A group of angiopoietin-1 receptor (Tie2)-positive NP cells was identified as potential key markers of NP progenitors and for future therapies [75]. Recent studies have found that Tie2^+^ NP progenitors decreased after IVDD induced by injury [59] and displayed an age-related pattern. In an in vitro study utilizing human IVDs, the majority of NP progenitors that highly expressed Tie2 were from donors below 20 years old, while NP progenitors from donors above 25 years old demonstrated much lower Tie2 expression [67]. Additionally, the loss of Tie2^+^ NP progenitors could be rejuvenated by MSC transplantation [76]. When human normal NP (NNP) and degenerated NP (DNP) were compared by single-cell RNA sequencing, five types of chondrocytes were found. Chondrocytes 1, staying in the starting position of the development trajectory, were dominant in NNP. Chondrocytes 2, 4 and 5 presented the activities of calcification inhibition, inflammation and calcifying, respectively. Chondrocytes 5 were thought to be key cells leading to NP degeneration, while chondrocytes 2 may play a role in delaying NP degeneration. In short, compared to NNP, DNP contained more chondrocytes that were in the later positions of the development trajectory, and chondrocytes responsible for NP degeneration, pain and inflammation increased in DNP [77]. In a study performed to compare BMSCs and NP progenitors, no significant difference was detected in the expansion ability of the two types of cells [78]. Additional evidence illustrates that UCSCs possessed an extensively higher proliferation ability than NP progenitors [49].

When referring to AF progenitor cells, different descriptions of their outlines, such as cobblestone-like and pancake-like, were used due to different cell sources and medium ingredients. CD29, CD44, CD69, CD105, Gata2 and Tnfaip3 were found to be positive, while CD34 and CD45 were negative. Three stemness markers, Oct4, SSEA4 and nucleostemin, were also discovered [17,79,80,81]. In addition, Scx was identified as one of the earliest markers of tendon progenitors, which were identical to AF progenitors in the early stages of development [82]. Multi-differentiation ability was also observed in AF progenitors, as adipocytes, osteocytes and chondrocytes can all be induced in appropriate cultures [80]. AF progenitors could also express type-I, II collagen and aggrecan, with biochemical tests showing accordant results [83]. Evidence indicating that chondroid matrix restoration is parallel to the reorientation and reestablishment of fibers in AF lamellae supports this finding [17]. However, no recent in vivo animal studies nor experiments have been conducted on impaired IVD models. 

CEP progenitors, which could differentiate into NP cells, could be enhanced by exosomes via the HIF1-α and TGF-β pathways [84]. Moreover, the exosomes of CEP progenitors could transport Sphk2 to NP cells and inhibit apoptosis [30]. When CEP progenitors were in the same coculture system, activating ERK1/2 and Akt pathways enhanced NP cell growth [29]. In vivo studies have verified the regeneration potential in rat [28,30] models. However, in a study that simultaneously covered NP, AF and CEP progenitors, the proliferation ability measured by both the cell growth curve and colony forming displayed no significant difference among them. In the study, CEP progenitors demonstrated the most powerful invasion ability over the other two cells [7], and they regenerated impaired IVD; this effect may have come from the exosomes produced by CEP progenitors, which penetrated the AF and migrated into NP cells [30]. They also restored the disc height and hydration in rat tail nucleotomy models and increased ECM protein levels, such as aggrecan and type-II collagen [85]. The same study revealed that the injected CEP progenitors preserved endogenous NP progenitors and exerted anti-inflammatory and anti-catabolic effects in impaired IVDs.

With certain stimulation, dermal fibroblasts originating from induced pluripotent stem cells (iPSCs) can differentiate into primitive streak (PS) mesoderm cells and then into NCs; these NCs maintain their phenotypes for at least 8 weeks and exert protective effects both in vitro and in vivo [86]. Human embryonic stem cells (hESCs) can also be induced to a similar notochord-NP cell lineage as iPSCs [87]. These findings were supported by transcriptomic similarities between the induced and native NP tissues and between the differentiating trajectories of iPSCs and hESCs [87]. In vivo studies conducted in rats showed that iPSCs-derived mature NP cells exerted a similar protective effect to induced NCs, possibly by replacing endogenous NP tissue spatially and functionally and preventing CEP destruction [88]. Because of their pluripotency, hESCs and iPSCs possessed greater differentiation ability than IVD progenitors and MSCs. However, the safety of hESCs and iPSCs still requires further validation.

## 5. Conclusions

Recent evidence from single-cell RNA sequencing provides more solid proof of the existence of IVD progenitors and the heterogeneity within the IVD. The early development of IVD progenitors is controlled by signaling pathways such as Wnt, BMP and retinoic acid. In addition, IVD progenitors may have advantages over MSCs because of their similarity with endogenous tissues, but the evidence for determining the optimal cell source for IVD regeneration is still lacking. In summary, progenitor cell-based therapy holds significant potential in repairing the degenerated IVD, and well-designed experiments are necessary to verify their therapeutic ability.

## Figures and Tables

**Table 1:** Isolation protocols of IVD progenitors from different tissues.

Source	Species	Digestion	Expansion	Function	Phenotype	Ref
NP	Human	Collagenase and pronase	αMEM with 10% FBS	CFU-S adolescent > older (25%) (limited)	Tie2^+^, GD2^+^	[11]
NP	Rat	0.25% type II collagenase	MSC complete medium	Colony-forming ability and multipotency	CD44^+^, CD73^+^, CD90^+^, CD105^+^, Sox2^+^, Nanog^+^, Oct4^+^	[13]
CEP	Rabbit	0.25% EDTA-trypsin and 0.2% type II collagenase	DMEM/F12 medium with 20% FBS	Multipotency	CD90^+^, CD105^+^, ACAN, Sox9^+^, Col2A^+^	[14]
CEP (degenerated)	Human	0.25% type II collagenase	DMEM/F12 medium with 10% FCS	Osteogenic Chondroblastic	CD73^+^, CD90^+^, CD105^+^	[15]
AF	Rabbit	Type I and II collagenase (150 U/mL)	αMEM with 15% FBS	Proliferation	iAF: Col2a1, Acan oAF: Col1a1	[16]
AF	Mouse	Collagenase P	DMEM/F12 medium with 10% FBS	Fibrogenic Chondrogenic	CD44^+^, Col1a1^+^, Col2a1^+^	[17]

**Table 2:** Comparisons of the characteristics of MSCs and disc progenitor cells.

	Phenotype	Differentiation	Expansion	Therapeutic Potential	Animal Models
BMSC	CD29^+^, CD90^+^, CD105^+^, CD146^+^	Osteocytes Adipocytes Chondrocytes NP cells AF cells	BMSC = NP progenitor	(1) IVD matrix promotion (2) Local cell regeneration (3) Differentiation (4) Immunomodulation	Rabbit Canine Porcine Rat Sheep
UCSC	CD29^+^, CD44^+^, CD73^+^, CD90^+^, CD105^+^, CD166^+^	Osteocytes Adipocytes Chondrocytes NP cells	UCSC < NP progenitor	(1) IVD matrix promotion (2) Local cell regeneration (3) Differentiation (4) Immunomodulation	Rabbit Rat
ADSC	CD73^+^, CD105^+^, CD44^+^, Sca-1^+^	Osteocytes Adipocytes Chondrocytes NP cells	/	(1) IVD matrix promotion (2) Local cell regeneration (3) Differentiation (4) Immunomodulation	Rabbit Mice Rat Sheep
NP progenitor	CD24^+^, CD44^+^, CD55^+^ CD70^+^ CD73^+^, CD82^+^, CD90^+^, CD105^+^, Tie2^+^, UTS2R^+^, PDGFRA^+^, KRT15^+^, Col2a1^+^, Col1a1^+^, Sox4^+^, LepR^+^	Osteocytes Adipocytes Chondrocytes NP cells CEP cells	NP progenitor = BMSC NP progenitor > UCSC (NP progenitor = AF progenitor = CEP progenitor)	(1) IVD matrix promotion (2) Local cell regeneration (3) Differentiation (4) Immunomodulation	Rat
AF progenitor	Scx^+^, Oct4^+^, SSEA4^+^, neucleostemin^+^, Col2^+^ CD29^+^, CD44^+^, CD69^+^, Grem1^+^, CD105^+^, Gata2^+^, Tnfaip3^+^, Car1^+^, LepR^+^	Osteocytes Adipocytes Chondrocytes AF cells	(NP progenitor = AF progenitor = CEP progenitor)	(1) IVD matrix promotion (2) Differentiation	
CEP progenitor	CD29^+^, CD44^+^, CD73^+^, CD90^+^, CD105^+^, CCNL1^+^, WSB1^+^	Osteocytes Adipocytes Chondrocytes NP cells CEP cells	(NP progenitor = AF progenitor = CEP progenitor)	(1) IVD matrix promotion (2) Local cell regeneration (3) Differentiation (4) Immunomodulation	Rat

## Data Availability

Not applicable.
